# Lung-Centric Inflammation of COVID-19: Potential Modulation by Vitamin D

**DOI:** 10.3390/nu13072216

**Published:** 2021-06-28

**Authors:** Hana. M. A. Fakhoury, Peter R. Kvietys, Ismail Shakir, Hashim Shams, William B. Grant, Khaled Alkattan

**Affiliations:** 1 Department of Biochemistry and Molecular Medicine, College of Medicine, Alfaisal University, P.O. Box 50927, Riyadh 11533, Saudi Arabia; 2 Department of Physiology, College of Medicine, Alfaisal University, P.O. Box 50927, Riyadh 11533, Saudi Arabia; pkvietys@alfaisal.edu (P.R.K.); ishakir@alfaisal.edu (I.S.); hshams@alfaisal.edu (H.S.); 3 Sunlight, Nutrition, and Health Research Center, P.O. Box 641603, San Francisco, CA 94164-1603, USA; wbgrant@infionline.net; 4 Department of Surgery, College of Medicine, Alfaisal University, P.O. Box 50927, Riyadh 11533, Saudi Arabia; KKattan@alfaisal.edu

**Keywords:** acute respiratory distress syndrome (ARDS), coronavirus, COVID-19, cytokine storm, inflammasome, neutrophil extracellular traps (NETs), SARS-CoV-2, vitamin D

## Abstract

SARS-CoV-2 infects the respiratory tract and leads to the disease entity, COVID-19. Accordingly, the lungs bear the greatest pathologic burden with the major cause of death being respiratory failure. However, organs remote from the initial site of infection (e.g., kidney, heart) are not spared, particularly in severe and fatal cases. Emerging evidence indicates that an excessive inflammatory response coupled with a diminished antiviral defense is pivotal in the initiation and development of COVID-19. A common finding in autopsy specimens is the presence of thrombi in the lungs as well as remote organs, indicative of immunothrombosis. Herein, the role of SARS-CoV-2 in lung inflammation and associated sequelae are reviewed with an emphasis on immunothrombosis. In as much as vitamin D is touted as a supplement to conventional therapies of COVID-19, the impact of this vitamin at various junctures of COVID-19 pathogenesis is also addressed.

## 1. Introduction

The mechanism of infection, transmission, and clinical presentations of SARS-CoV-2 are qualitatively similar to those of its predecessor, SARS-CoV [1,2,3]. Notably, both SARS-CoV-2 and SARS-CoV highjack angiotensin-converting enzyme 2 (ACE2) on the membranes of host cells to gain entry [4]. ACE2 is expressed on apical membranes of human respiratory and gastrointestinal epithelial cells [5,6], accounting for proposed means of transmission and clinical manifestations of the current disease, COVID-19. Specifically, human-to-human transmission of SARS-CoV-2 may occur via expired air droplets or a fecal–oral route; the former well documented [3], while the latter has been posited [7]. The clinical presentations include respiratory problems (e.g., cough, dyspnea) and to a lesser extent intestinal complaints (e.g., diarrhea) [8,9]. Most infected individuals develop mild symptoms that resolve without the need for hospitalization. However, a small number of patients develop pneumonia and require hospitalization, and eventually recover. Others worsen, progressing to acute respiratory distress syndrome (ARDS) and require aggressive treatment in intensive care units. The major cause of death is respiratory failure [10,11,12,13], although multiple organ damage and sepsis can occur in severe COVID-19 cases [2,11]. In accordance with the clinical presentation, microscopic analyses of autopsy specimens indicate that lungs bear the greatest pathologic burden [10,14,15,16,17]. The predominant histologic features include diffuse alveolar damage, thrombosis, and inflammatory infiltrates consisting of macrophages, lymphocytes, and neutrophils [10,15,16,17,18,19,20,21,22,23]. Organs remote from the initial site of infection (e.g., heart, kidney, brain) may also exhibit pathology (e.g., thrombi) [10,14,17,24,25]. Of particular significance, thrombi, macrophage recruitment, and diminished T and B lymphocytes are noted in hilar lymph nodes and the spleen [15].

The mechanisms involved in the pathogenesis of COVID-19 are the subject of an intense research effort. The information emerging indicates that, in addition to viral virulence, the host’s immune response appears to play a major role. Specifically, an excessive inflammatory response, coupled with an impaired antiviral (e.g., interferon) response, are currently touted as causative [26,27,28,29,30,31,32,33,34]. A common characteristic of severe COVID-19 patients is lymphopenia; markers of T cell exhaustion are also reported in some [15,27,28,30,33,35,36,37,38,39,40,41]. An inadequate interferon response would impede the eradication of the virus thereby exacerbating and prolonging the inflammatory response and associated sequelae [27,28,29,42]. As a case in point, a significant contribution to the lethality of COVID-19 is the inflammation-induced formation of microvascular thrombi, referred to as immunothrombosis [43,44,45,46,47,48,49].

Vitamin D can impact numerous pathways involved in host immune responses to viral infections [50,51]. The dietary or skin-derived vitamin D precursors are sequentially hydroxylated to form the active vitamin D [1,25(OH)2D]. A variety of immune and non-immune cells possess the enzymatic machinery to generate (e.g., CYP27B1) or inactivate (e.g., CYP24A1) vitamin D [50,52,53,54,55]. Vitamin D can exert both genomic and non-genomic effects by binding to the vitamin D receptor (VDR). A well-documented genomic function is the generation of the antiviral peptide, LL-37 [50,51,56]. A unique regulatory feature of vitamin D is the ability to induce an appropriate inflammatory response and suppress an excessive one [51].

A significant majority of COVID-19 patients have vitamin D insufficiency, based on cut-off values for 25-OHD ≤ 10–20 ng/mL [57,58,59,60,61]. A recent meta-analysis indicates that low levels of vitamin D (20–30 ng/mL) are associated with a greater susceptibility to SARS-CoV-2 infection, as well as severity and mortality of COVID-19 [62]. Conversely, in a multicenter retrospective study, supplemental 25-OHD during the first month of hospitalization reduced in-hospital mortality [63]. Thus, it is not surprising that vitamin D has been touted as a potential therapeutic adjunct to conventional approaches in the treatment of COVID-19 [56,57,60,64]. However, based on some controversial issues [52], caution is recommended pending the outcomes of clinical trials targeting the therapeutic efficacy of vitamin D in COVID-19 patients [60,64]. 

**Figure 1:**
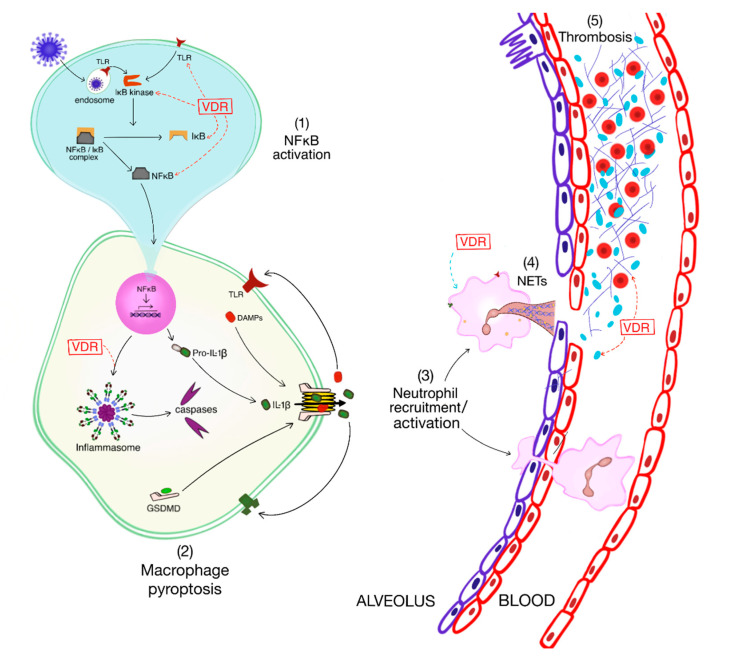
Impact of vitamin D/VDR signaling at various junctures of lung-centric inflammation and immunothrombosis. Experimentally verified inhibitory pathways are indicated with red dashed arrows while an ambiguous pathway is indicated with a blue dashed arrow. As addressed in the text, VDR signaling inhibits the response of alveolar macrophages to viral infection at the level of (1) NFκB signaling and (2) inflammasome activation, thereby preventing neutrophil recruitment and activation (3). The impact of VDR signaling on NET generation (4) may be context-dependent; VDR can promote or inhibit NETs. VDR signaling inhibits thrombosis (5) by interfering with platelet function and fibrin generation. VDR: Vitamin D Receptor; NETs: Neutrophil Extracellular Traps.

Herein, a narrative approach will be used to address the role of SARS-CoV-2 in lung inflammation that can lead to the manifestations of severe COVID-19 such as ARDS, coagulopathy, and multiorgan dysfunction. The potential impact of vitamin D at various stages of COVID-19 pathogenesis will be addressed in an evidence-based manner (Figure 1). To this end, the PubMed database was mined for vitamin D/VDR data relevant to the aberrant immune response of COVID-19 and yielded the following. Studies addressing the prophylactic or therapeutic efficacy of vitamin D for the ARDS of COVID-19 are limited [57,63,65,66,67]. A transgenic murine model of COVID-19 that mimics the disease in humans is available [68,69]; however, it has not been used for interventional studies of vitamin D/VDR signaling. An additional issue complicating animal studies is the potential for species-specific inflammatory signaling pathways [50]. Finally, the bulk of the information is derived from cell-based studies using tractable immune cells (e.g., circulating monocytes), which may not reflect the responses in relevant lung cells (e.g., alveolar macrophages). With these limitations in mind, we address the most salient features of vitamin D/VDR signaling relevant to the innate immune response of ARDS. Wherever possible, reviews are cited to direct the reader to relevant original studies. 

## 2. Current Status of Knowledge 

### 2.1. Intrapulmonary Tropism 

SARS-CoV-2 productively infects the human nasal and bronchiolar epithelial cells; primarily ciliated epithelia and, to a lesser extent, goblet cells [4,5,12,20,70,71,72,73]. The initial event is an adhesive interaction between the spike (S) protein of the virus with ACE2 on apical membranes of lung epithelia. Subsequently, the S protein is proteolytically activated (e.g., TMPRSS2, furin) allowing for fusion-induced entry [4,74]. After replicating its genome, SARS-CoV-2 preferentially exits via the apical membrane [20,71]. This mode of entry and exit would ensure infection of downstream lung epithelial cells while limiting remote organ involvement. The epithelium remains intact for up to 2–4 days post infection (dpi); progressive infection eventually results in epithelial permeability [71,72]. Of note is that little injury is incurred by SARS-CoV-2 replication within epithelial cells until 3–4 dpi, after which epithelial cell injury and death occur [70,71,72]. Cell death is a result of apoptosis [71,72], presumably as an antiviral defense mechanism [75,76]. 

Within the alveolar compartment, type II epithelial cells, endothelial cells, as well as macrophages and dendritic cells, express the requisite machinery for SARS-CoV-2 infection (e.g., ACE2, TMPRSS2, and/or furin) [10,15,26,77]. Infection of type II epithelial cells is productive and utilizes an apical entry and exit pathway [78]. Infection results in the upregulation of pro-inflammatory and antiviral transcriptional pathways [73,78]. The pro-inflammatory pathways (e.g., NFκB) are dominant in the early stages of infection (1–2 dpi); whereas antiviral interferon signaling (e.g., STAT) is delayed (3–4 dpi). As the infection progresses, apoptotic pathways become activated [73,78]. Loss of type II pneumocytes is particularly detrimental, since they generate surfactant, reabsorb fluid from the airspace, and serve as progenitors for the repair of epithelial damage [79]. 

SARS-CoV-2 infection of endothelium is a matter of debate. Capillary organoids are permissive for SARS-CoV-2 infection and replication [80]. However, lung autopsies of COVID-19 patients are equivocal; some report infection of the endothelium [10], while others highlight the lack of endothelial infection [18,19]. Irrespective of this, endothelial dysfunction and/or injury is common, as evidenced by microvascular thrombi, inflammatory cell infiltration, and capillary sprouting [10,18,19]. A probable scenario holds that endothelial dysfunction contributes to the formation of occlusive emboli resulting in hypoxia, a powerful stimulus for angiogenesis [10]. 

The major sentinel immune cells of the lung are the alveolar macrophages and dendritic cells. There seems to be little doubt that macrophages can be infected by SARS-CoV-2 [12,24,26,77,81,82,83]. However, as compared to pneumocytes, fewer resident or infiltrated macrophages are infected [18,19]. While phenotypically quite diverse [84], alveolar macrophages are generally classified as either pro-inflammatory (M1) or pro-resolving (M2) [77]. SARS-CoV-2 can infect both M1 and M2 macrophages; M1 being more permissive [82]. Bronchoalveolar lavage fluid (BALF) of severe COVID-19 patients is characterized by a diminished resident M2 population in favor of infiltrated M1 macrophages [85]. Of note is that SARS-CoV-2 infection of macrophages does not yield viable progeny [81,82]. Despite an abortive infection, the macrophages can generate pro-inflammatory cytokines/chemokines. Dendritic cells, major antigen-presenting cells, can also be infected by SARS-CoV-2. As was the case in macrophages, the infection is abortive [81]. Further, their interferon response is diminished; an effect attributed to viral antagonism of signaling pathways (e.g., STAT). Dendritic cells isolated from COVID-19 patients exhibit impaired maturation and functionality, as evidenced by an inability to stimulate CD4 and CD8 T cell proliferation [37].

In summary, SARS-CoV-2 can productively infect nasal, bronchial, and alveolar epithelial cells, while infection of macrophages and dendritic cells is abortive. This cell-specific differential infection (productive vs. abortive) is the same as noted with SARS-CoV [77]. Since nearly peak viral titers are incurred prior to discernible cytopathic effects, epithelial cell death (primarily, apoptosis) is not considered to be a direct effect of the virus; rather, it is attributed to the host immune response [10,70,73,78]. The net effect of SARS-CoV-2 induced alveolar damage (epithelial and endothelial) is a breakdown of the air-blood barrier, thereby limiting oxygen exchange and eventually culminating in respiratory failure. 

### 2.2. Intrapulmonary Tropism: Impact of Vitamin D

Apart from being a receptor for SARS-CoV-2, ACE2 has a homeostatic function in the lungs by regulating the local renin–angiotensin system (RAS) [86,87]. In brief, renin-derived angiotensin I (AngI) is converted to angiotensin II (Ang II) by ACE. Ang II interacts with its receptor (AT1R) which triggers downstream pathways that are detrimental to lung function (e.g., pro-oxidant, pro-inflammatory). As a countermeasure, ACE2 nullifies the effects of Ang II by cleaving it to the heptapeptide Ang 1–7 which interacts with its cognate receptor (MasR) to exert beneficial effects (e.g., antioxidant, anti-inflammatory). Thus, an imbalance in the relative activity of the two converting enzymes that favor ACE over ACE2 promotes lung injury and vice-versa. Vitamin D is a negative regulator of the local RAS (increasing ACE2/ACE ratio) and thereby protects against acute lung injury (ALI) in rodents. For example, ALI induced by local (LPS, acid) or remote (peritonitis) challenges increases lung inflammation and injury as well as systemic hypoxia; effects attributed to an increased local RAS and ACE/ACE2 ratio [88,89,90,91]. Vitamin D/VDR signaling suppresses lung inflammation and injury by inhibiting Ang II/AT1R signaling and promoting Ang 1–7/MasR signaling [88,90]. Based on these and other documented effects of vitamin/VDR signaling on the local RAS, vitamin D has been touted as a potential therapeutic approach to treat ARDS of COVID-19 [92]. As a caveat, SARS-CoV-2 uses ACE2 to infect human lung cells [4,74,78]. Thus, it is unclear how a vitamin D-induced increase in the ACE2/ACE ratio will impact lung injury or disease progression induced by SARS-CoV-2. 

At mucosal sites exposed to the external environment (e.g., gut, bronchi), antimicrobial peptides (AMPs) represent the first line of defense against pathogens [53,93]. LL37 is an AMP that can be detected in isolated lung epithelial cells and alveolar macrophages [94]. The cathelicidin gene encoding LL37 contains a vitamin D response element and can be regulated by vitamin D/VDR signaling [50]. Human bronchial epithelial cells constitutively express the requisite machinery (e.g., CYP27B1, VDR) to ensure intracrine activation of vitamin D/VDR signaling in response to the exogenous inactive vitamin D precursor, 25(OH)D. VDR-induced transcription generates LL37 in isolated human airway epithelial cells even in the absence of infection; however, viral infection has a potentiating effect [54,95,96,97]. The antiviral effects of LL37 include both extracellular (e.g., destruction of the viral envelope) and intracellular (e.g., inhibition of viral replication) modalities [54]. Based on its broad antiviral activity, it has been proposed that the vitamin D–LL37 axis may be effective against SARS-CoV-2 [56]. LL37 may also inhibit binding of SARS-CoV-2 to ACE2. In silico structural studies predict binding sites for LL37 on the viral S protein [98] and in a cell-free system this interaction prevents the binding of the S protein to ACE2 [99]. Of note, in a small safety and efficacy trial in COVID-19 patients, oral administration of *L. lactis*, genetically modified to produce LL37, was deemed safe and alleviated respiratory symptoms such as cough and shortness of breath [100]. However, the enrolled cohort were only mildly symptomatic and firm conclusions of therapeutic efficacy await controlled larger scale clinical trials. 

COVID-19 lung histopathology is characterized by inflammatory cell infiltration and diffuse alveolar damage, with the blood–air barrier defect ultimately causing systemic hypoxia. In general, neither dietary depletion, genetic blockade, nor supplementation of vitamin D appreciably affects the inflammatory status or epithelial integrity of the unstressed lungs of rodents [88,90,101,102,103,104]. However, in models of ALI (e.g., LPS), either vitamin D or VDR deficiency exacerbates lung inflammation, barrier dysfunction, and systemic oxygenation [102,104]; meanwhile, supplementation with vitamin D is protective [88,104,105,106]. Although there are detractors from this paradigm [101,107], these detractors may not be anomalies. Seemingly paradoxical roles of vitamin D are most likely context-dependent (e.g., species, models, cell types) [54,55,96].

A context-dependent vitamin D/VDR signaling is also operative in the immune sentinel cells of the lung, such as alveolar macrophages and dendritic cells. A common cell-based model employs either bone marrow- or monocyte-derived macrophages and dendritic cells. Ex vivo induction of human macrophage differentiation in the presence of vitamin D does not appear to affect their polarization to either M1 or M2 phenotypes [108,109]. Further, vitamin D/VDR signaling in differentiated macrophages is either pro- or anti-inflammatory depending on the existing infectious/inflammatory milieu [108,109,110,111,112,113,114]. Consensus holds that, in response to viral infection, macrophage vitamin D/VDR signaling initially activates pro-inflammatory pathways (e.g., increased LL37, IL-8), while a more delayed anti-inflammatory response (e.g., decreased IL-8, increased IL-10) serves to limit immune-mediated injury [54,55,112,114]. With respect to human dendritic cells, supplementation with vitamin D during or after differentiation renders them tolerogenic [53,55,115,116]. Tolerogenic dendritic cells generate an anti-inflammatory milieu by secreting less pro-inflammatory cytokines, inhibiting effector T cell function (both CD4+ and CD8+ T cells), and promoting regulatory T cell conversion [55,115,117]. The vitamin D-induced tolerogenic response is delayed, presumably due to a delay in upregulation of CYP27B1 and VDR expression in both dendritic and effector T cells. It has been proposed that this delay allows for the clearance of invading microbes and subsequently quiets the immune response to avoid collateral tissue damage [55].

### 2.3. The Inflammatory Response 

Transcriptomic [118] and proteomic [33,40,85,119,120] analyses of bronchoalveolar lavage fluid (BALF) of COVID-19 patients indicate that a pro-inflammatory environment is present in their lungs. Their BALF contains high levels of chemokines and cytokines, with the former detected earlier in longitudinal sampling [85]. Correspondingly, the BALF was enriched with innate immune cells such as neutrophils, monocytes, and to a lesser extent, dendritic cells [33,40,85,119]. The neutrophils and macrophages exhibited an activated phenotype in comparison to their circulating counterparts. The generation of a pro-inflammatory milieu is most likely initiated by either infected epithelial cells or resident macrophages [18,19,119]. These cells detect specific molecular features of inhaled virions (e.g., RNAs, proteins) referred to as pathogen-associated molecular patterns (PAMPs). Different PAMPs are recognized by an array of pattern recognition receptors (PRRs) that activate various signaling pathways, most of which converge to activate the transcription factor, NFκB [26,27,78]. NFκB transactivates various pro-inflammatory genes, generating chemokines (e.g., IL-8) and cytokines (e.g., IL-6, TNFα) [26,27,78,121]. As the infection progresses, leading to tissue injury [15], PRRs on macrophages recognize material released by damaged cells (damage-associated molecular patterns; DAMPs) and mount signaling cascades that also activate NFκB and thereby amplify the inflammation. The PRR/NFκB pathway has been proposed as a potential therapeutic target for COVID-19 [122,123].

A major function of NFκB is to initiate the assembly and activation of the NLRP3 inflammasome; a multiprotein complex that generates IL-1β. This cytokine lacks a signal sequence, so secretion to extracellular space occurs through pores in the plasma membrane formed by gasdermin D (GSDMD) [124,125]. Multiple GSDMDs are inserted into the plasma membrane and oligomerize to form pores [126], thereby allowing IL-1β to leave the cell. Excessive GSDMD pores can rupture the plasma membrane and induce a lytic form of cell death, termed “pyroptosis” [124,125]. 

Emerging evidence indicates that an NLRP3 inflammasome is formed in COVID-19 patients and may predict the disease trajectory. Human monocytes infected by SARS-CoV-2 secrete IL-1β and undergo pyroptosis [127,128], indicating that the virus can induce a functional inflammasome. Sera of COVID-19 patients contain active caspase; higher levels are prevalent in more severe cases [127]. Furthermore, lung tissues of fatal cases contain the fully assembled NLRP3 inflammasome [127,129]. The inflammasome components are localized to the resident or recruited monocytes/macrophages and, to a lesser extent, alveolar epithelial cells. It has been proposed that the enhanced lethality of COVID-19 in older patients is a result of age-related hyperactivation of the NLRP3 inflammasome [130]. 

### 2.4. The Inflammatory Response: Impact of Vitamin D 

A major family of PRRs are the toll-like receptors (TLRs), membrane spanning glycoproteins that can detect viral PAMPs [131] and host DAMPs [122]. SARS-CoV-2, like other coronaviruses, are most likely sensed via their nucleic acids (e.g., ssRNA, dsRNA) by endosomal PRRs (e.g., TLR3 and TLR7). In addition, viral membrane proteins as well as various DAMPs from injured cells can be detected by plasma membrane PRRs (e.g., TLR4). Agonists of TLR3 and TLR4 increase cytokine (e.g., TNFα, IL-1β) production by human lung macrophages, with TLR4 agonists being the most potent [132]. In human monocytes, vitamin D/VDR signaling reduces surface levels of TLR4, while not affecting intracellular TLR3 [133,134]. Thus, while the vitamin D/VDR axis may not impact viral-mediated TLR signaling, it may downregulate DAMP-mediated TLR pathways.

In quiescent cells, NFκB is inhibited by IκB which binds to the NFκB dimer and prevents its translocation to the nucleus [121]. Pro-inflammatory stimuli activate IκB kinase which phosphorylates IκB. Subsequent ubiquitination targets IκB for proteasomal degradation. The loss of IκB frees the NFκB dimer to enter the nucleus and transcribe relevant pro-inflammatory genes. Several lines of evidence indicate that the nuclear translocation of NFκB is impeded by vitamin D/VDR signaling. In VDR deficient fibroblasts, there is a reduction in basal levels of IκB and an increase in nuclear levels of NFκB [135]. Exogenous vitamin D increases IκB and decreases NFκB translocation to the nucleus of human lung epithelial cells or murine macrophages [136,137]. In a similar vein, vitamin D or VDR overexpression inhibits IκB kinase activity in fibroblasts; an effect mediated by the physical interaction of the VDR with the kinase [138]. Finally, VDR can also physically interact with NFκB, as demonstrated in murine tissues [139,140] and macrophages [141]. However, the precise docking sites involved in VDR interactions with IκB kinase and NFκB have not been identified. 

Vitamin D/VDR signaling inhibits tissue inflammation and injury mediated by the NLRP3 inflammasome in various in vivo murine models [142,143,144]. Loss and gain of function approaches support a role for the vitamin D/VDR pathway to dampen activation and the function of the NLRP3 inflammasome. For example, VDR inhibits caspase activation, generation of mature IL-1β, and GSDMD-mediated pyroptosis in a murine model of kidney injury, as well as human tubular epithelial cells [143,144]. VDR can physically interact with NLRP3 [144,145]; the ligand-binding domain of VDR and the amino-terminal pyrin domain of NLRP3 are required for complex formation [144]. The VDR-NLRP3 interaction prevents the inflammasome function in both murine and human macrophages [144]. 

An increase in intracellular oxidant stress has been implicated in the activation of the NLRP3 inflammasome [146,147,148]. Oxidant stress occurs when the generation of ROS exceeds the antioxidant capacity of the cell. An important transcription factor that enhances the antioxidant status of cells is Nrf2 [149]. The promoter region of the Nrf2 gene contains a response element that binds VDR [150]. In human epithelial cells, vitamin D/VDR signaling blunts ROS-mediated activation of the NLRP3 inflammasome by promoting Nrf2 translocation to the nucleus where its transcriptional activity increases cellular antioxidant enzymes [151]. 

### 2.5. Immunothrombosis and Remote Organ Dysfunction

Organs remote from the initial site of SARS-CoV-2 infection can exhibit pathology, particularly in severe cases [2,10,14,15,17,24,83,152]. It has been proposed that the excessive inflammatory response within the lungs results in the spill-over of cytokines into the systemic circulation causing a “cytokine storm” syndrome [12,26,29,153,154]. However, a more likely scenario is the generation of a localized cytokine storm within the lungs of COVID-19 patients [120,155].

The subsequent recruitment and hyper-activation of neutrophils results in the generation of neutrophil extracellular traps (NETs) [156,157]. NET components have been detected in tracheal aspirates [43,158] of COVID patients. Lung tissue from fatal cases contains NETs in close association with diffuse alveolar damage [43,44,158]. Importantly, NETs decorated with platelets as well as occlusive thrombi have been noted within the lung microvasculature [43,44,45]. These observations are in accordance with immunothrombosis, a process linking innate immunity to thrombosis for defense against pathogens [23,49,159]. However, excessive immunothrombosis leads to occlusion of numerous pulmonary blood vessels and precipitates ARDS.

Whether the immunothrombosis of COVID-19 is confined to the lungs or can impact remote organs is still controversial. Activated neutrophils and platelets, as well as platelet–neutrophil aggregates, are present in the systemic circulation of patients [45,160]. Further, sera from COVID-19 patients can induce NET formation in neutrophils isolated from healthy donors [161]. Circulating markers of fibrin degradation (e.g., D-dimers), and NET remnants are elevated in COVID-19, with higher levels in more severe or fatal cases [45,161]. However, while NET formation has been consistently noted in the lungs of COVID-19, their presence in remote organs has either been noted [45] or not detected [44]. Nonetheless, in fatal cases of COVID-19, microvascular thrombi as well as ischemic infarcts are present in multiple organs [10,14,17,24,152].

### 2.6. Immunothrombosis and Remote Organ Dysfunction: Impact of Vitamin D

While clinical studies indicate an inverse relationship between vitamin D status and thrombotic events [162], the impact of VDR signaling on specific steps involved in the development of immunothrombosis is less clear. For example, the effects of vitamin D/VDR signaling on the generation of NETs is ambiguous [163,164]. Platelet activation is increased in blood samples from vitamin D deficient individuals [165], while vitamin D inhibits platelet aggregation in vitro [166]. The antithrombin gene has multiple vitamin D response elements, and paricalcitol increases antithrombin expression in, and secretion from, cultured cells [167]. Further, a transcriptomic analysis of data derived from human monocytes identified the thrombomodulin gene as a target of vitamin D/VDR signaling [168]. Collectively, these observations predict an antithrombotic function of vitamin D/VDR signaling. Unexpectedly, however, correcting vitamin D deficiency in otherwise healthy individuals does not consistently affect their blood thrombogenic profile. Vitamin D supplementation of deficient subjects either increases [169] or reduces [170] thrombogenicity. 

Further work is warranted to systematically assess the potential benefit of vitamin D in immunothrombosis of COVID-19. This is particularly important since anticoagulants (e.g., heparinoids) are currently advocated to alleviate hypercoagulation in these patients [171] and therapeutic vitamin D may increase the probability of bleeding events [172]. 

## 3. Conclusions

The anti-inflammatory and anti-thrombotic effects of vitamin D are promising features that suggest efficacy against immunothrombosis of COVID-19. Results of ongoing clinical trials should either validate or refute a beneficial role for vitamin D in alleviating the ARDS of COVID-19 and associated sequelae. 

## Data Availability

Not applicable.
